# Adaptive neural network control for uncertain dual switching nonlinear systems

**DOI:** 10.1038/s41598-022-21049-y

**Published:** 2022-10-05

**Authors:** Qianqian Mu, Fei Long, Lipo Mo, Liang Liu

**Affiliations:** 1 College of Big Data and Information Engineering, Guizhou University, Guiyang, 550025 Guizhou China; 2 School of Mathematics and Big Data, Guizhou Education University, Guiyang, 550018 Guizhou China; 3 School of Artificial Intelligence and Electrical Engineering, Guizhou Institute of Technology, Guiyang, 550003 Guizhou China; 4 School of Mathematics and Statistics, Beijing Technology and Business University, Haidian, 100048 Beijing China

**Keywords:** Applied mathematics, Computational science, Electrical and electronic engineering, Engineering

## Abstract

Dual switching system is a special hybrid system that contains both deterministic and stochastic switching subsystems. Due to its complex switching mechanism, few studies have been conducted for dual switching systems, especially for systems with uncertainty. Usually, the stochastic subsystems are described as Markov jump systems. Based upon the upstanding identity of RBF neural network on approaching nonlinear data, the tracking models for uncertain subsystems are constructed and the neural network adaptive controller is designed. The global asymptotic stability almost surely (GAS a.s.) and almost surely exponential stability (ES a.s.) of dual switching nonlinear error systems are investigated by using the energy attenuation theory and Lyapunov function method. An uncertain dual switching system with two subsystems, each with two modes, is studied. The uncertain functions of the subsystems are approximated well, and the approximation error is controlled to be below 0.05. Under the control of the designed adaptive controller and switching rules, the error system can obtain a good convergence rate. The tracking error is quite small compared with the original uncertain dual switching system.

## Introduction

Switched system is a special class of hybrid dynamical system composed of several dynamical subsystems and a switching law that specifies the active subsystem at each instant of time. Switched systems can be divided mainly into two categories based on the switching driving mechanism: deterministic switched systems and stochastic switched systems. Deterministic switched system is a switched system with controllable switching rules, such as automobiles with automatic transmissions and aircraft with attitudinal switching^1–3^. Stochastic switched system is usually described by a Markov jump system, which represents the randomness of the system structure or parameters.

With the growing complexity of modern engineering systems, it is difficult to describe such engineering systems using single switching systems. Dual switching system (DSS) was introduced as a framework for modeling hybrid systems^4^. It’s a new type of system with both deterministic and stochastic signals^5,6^. The research of dual switching systems has mainly focused on the stability of the systems. Song^7^ studied almost sure stability of switching Markov jump linear systems. Bolzern et al.^8^ proposed a model of dual switching discrete-time linear systems, and a stability strategy was determined. Yang et al.^9^ investigated the robust exponential almost sure stability of discrete-time two-level switched systems.

Some effective control schemes have been developed for uncertain nonlinear systems. Lopez-Sanchez^3^ studied an external control loop consisting of a robust online-learning generalized-regression neural network and presented trajectory analysis. Cheng^10^ proposed an adaptive neural network control approach to achieve accurate and robust control of nonlinear systems with unknown dynamics. Chen^11^ investigated the issue of developing an event-triggered adaptive tracking controller of a class of uncertain nonlinear systems. Tang^12^ studied an adaptive prescribed-performance control problem for a class of switched uncertain nonlinear systems under arbitrary switching signals. Lu^13^ investigated the resilient adaptive neural control for uncertain nonstrict-feedback systems with an infinite number of actuator failures. Wang^14–17^ has conducted research on adaptive control for nonlinear systems in recent years using event-triggered prescribed settling time consensus compensation control^14^, neural adaptive self-triggered control^15^, fuzzy adaptive event-triggered finite-time constraint control^16^ and adaptive neural sliding mode control^17^ methods, respectively. In conclusion, approximating the uncertain function using neural network in single systems or single switching systems is effective. In conclusion, approximating the uncertain function using neural network in single switching systems is effective. However, unlike the above literature, the switching mechanism of the research object in this paper is more complex. The difficulty in solving this problem is that the approximation system and the error system are also dual switching, which requires higher design requirements for the switching strategy and stricter conditions for stability.

Motivated by the above discussion, we propose an adaptive neural network control method for uncertain dual switching nonlinear systems. The main contributions of this paper are summarized as follows:

- A neural network adaptive control method based on radial basis functions is proposed. The radial basis function neural networks (RBFNNs) were trained based on historical data to estimate the unknown disturbances firstly. And then adaptive control law was designed to adjust the network weights by feedback error through the dynamic tracking process. This is different from previous approaches mentioned above.
- A deterministic switching strategy for dual switching system was designed. To address the randomness caused by the stochastic subsystem, the switching strategy was designed to determine the current active subsystem by using the tracking error expectation. This ensured that the deterministic switched subsystem that had the minimum error expectation was activated.
- Sufficient conditions for GAS a.s. and ES a.s. of the error tracking system are provided to guarantee stable tracking of the uncertain dual switching nonlinear system.

The rest of the article is structured as follows. In “Problem formulation”, the formulation of the problem and the preliminary results are given. The adaptive neural network controller design and the GAS a.s. and ES a.s. analysis is presented in “Results”. Finally, a simulation example is presented to prove the effectiveness of the proposed control scheme in “Simulation”.

## Problem formulation

The system description and some useful definitions are provided in this section.

**Figure 1:**
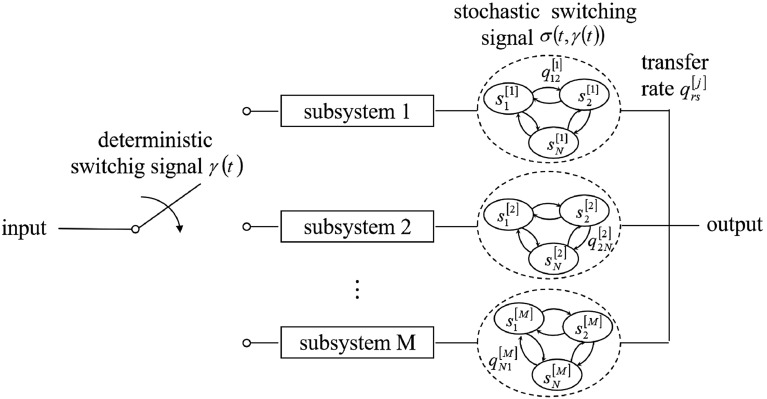
Structure of a DSS.

**System description**. Consider the following nth-order nonlinear differential equation:

1 $$\begin{aligned} {{y}^{\left( n \right) }}=f_{\sigma \left( t,\gamma \left( t \right) \right) }^{\left[ \gamma \left( t \right) \right] }\left( y,{{y}^{\left( 1 \right) }},\ldots ,{{y}^{\left( n-1 \right) }} \right) + g_{\sigma \left( t,\gamma \left( t \right) \right) }^{\left[ \gamma \left( t \right) \right] }\left( y,{{y}^{\left( 1 \right) }},\ldots ,{{y}^{\left( n-1 \right) }} \right) u_{\sigma \left( t,\gamma \left( t \right) \right) }^{\left[ \gamma \left( t \right) \right] }, \end{aligned}$$

where the system state $y\left( t \right) \in {{R}}$, the nonlinear funcitions $f_{\sigma \left( {t,\gamma \left( t \right) } \right) }^{\left[ {\gamma \left( t \right) } \right] } = f_\sigma ^\gamma \in R$ and $g_{\sigma \left( {t,\gamma \left( t \right) } \right) }^{\left[ {\gamma \left( t \right) } \right] } = g_\sigma ^\gamma \in R$ are unknown. The deterministic switching signal $\gamma \left( t \right) = {\gamma _k} \in M = \left\{ {1,2, \ldots ,m} \right\}$ for $t \in \left[ {{t_k},{t_{k + 1}}} \right)$ is a right continuous piece-wise constant function and represents the k-th deterministic subsystem is active . $\left\{ {{t_0},{t_1}, \ldots ,{t_k}} \right\}$ denotes the sequence of switching instants, and ${t_k}$ is the k-th switching time. The stochastic switching signal $\sigma \left( {t,\gamma \left( t \right) } \right) = \sigma \left( {{\tau _v},{\gamma _k}} \right) \in N = \left\{ {1,2, \ldots ,n} \right\}$ for $t \in \left[ {{\tau _v},{\tau _{v + 1}}} \right) \subset \left[ {{t_k},{t_{k + 1}}} \right)$ is a piece-wise constant function, which is right-continuous and governed by an N-mode Markov process and represents the v-th stochastic subsystem is active. $\left\{ {{\tau _0},{\tau _1}, \ldots ,{\tau _l}} \right\}$is the sequence of jump times within $t \in \left[ {{t_k},{t_{k + 1}}} \right)$ , and $u_{\sigma \left( {t,\gamma \left( t \right) } \right) }^{\left[ {\gamma \left( t \right) } \right] } = u_\sigma ^\gamma \in R$ is the control input. The structure of a DSS is illustrated by Fig. 1.

**Radial basis function neural networks**. RBF neural networks are capable of approximating any continuous function arbitrarily well^18^. The smooth functions $f_\sigma ^\gamma \left( x \right)$ and $g_\sigma ^\gamma \left( x \right)$ in (1) can be represented as

2 $$\begin{aligned}{}&{\hat{f}}_\sigma ^\gamma \left( x \right) = {\omega _{f_\sigma ^\gamma }}^T{\varphi _{f_\sigma ^\gamma }}\left( x \right) , \end{aligned}$$

3 $$\begin{aligned}{}&{\hat{g}}_\sigma ^\gamma \left( x \right) = {\omega _{g_\sigma ^\gamma }}^T{\varphi _{g_\sigma ^\gamma }}\left( x \right) , \end{aligned}$$

where $\omega _{f_\sigma ^\gamma }$ and $\omega _{g_\sigma ^\gamma }$ are corresponding weight vectors. ${\varphi _{f{{_\sigma ^\gamma }_i}}}\left( x \right)$ and ${\varphi _{g{{_\sigma ^\gamma }_i}}}\left( x \right)$ are Gaussian radial basis functions with the form of ${\varphi _i}\left( x \right) = \exp \left( {{{ - {{\left\| {x - {c_i}} \right\| }^2}} / {2{\sigma _i}^2}}} \right)$. For an arbitrary constant $\varpi$, $\left| {f_\sigma ^\gamma \left( x \right) - {\hat{f}}_\sigma ^\gamma \left( x \right) } \right| < \varpi$ and $\left| {g_\sigma ^\gamma \left( x \right) - {\hat{g}}_\sigma ^\gamma \left( x \right) } \right| < \varpi$. The approximation error is usually assumed to be upper bounded by $\varpi$^19^. In the reachable workspace of *x*, the weight optimization combination is computed by

4 $$\begin{aligned}{}&\omega _{f_\sigma ^\gamma }^* = \arg \mathop {\min }\limits _{{\omega _{f_\sigma ^\gamma }} \in {\Omega _{{w_f}}}} \left[ {\mathop {\sup }\limits _{x \in \Omega } \left| {f_\sigma ^\gamma - {\omega _{f_\sigma ^\gamma }}^T{\varphi _{f_\sigma ^\gamma }}\left( x \right) } \right| } \right] , \end{aligned}$$

5 $$\begin{aligned}{}&\omega _{g_\sigma ^\gamma }^* = \arg \mathop {\min }\limits _{{\omega _{g_\sigma ^\gamma }} \in {\Omega _{{w_g}}}} \left[ {\mathop {\sup }\limits _{x \in \Omega } \left| {g_\sigma ^\gamma - {\omega _{g_\sigma ^\gamma }}^T{\varphi _{g_\sigma ^\gamma }}\left( x \right) } \right| } \right] . \end{aligned}$$

Next, we define the RBF network approximation errors as

6 $$\begin{aligned} W_\sigma ^\gamma = {\hat{f}}_\sigma ^\gamma \left( x \right) - f_\sigma ^\gamma \left( x \right) + \left( {{\hat{g}}_\sigma ^\gamma \left( x \right) - g_\sigma ^\gamma \left( x \right) } \right) u_\sigma ^\gamma \end{aligned}$$

Combined with (2)–(3), the minimum network approximation error is computed by

7 $$\begin{aligned} \left( {{\varepsilon _{\min }}} \right) _\sigma ^\gamma = {\left( {\omega _{f_\sigma ^\gamma }^*} \right) ^T}{\varphi _{f_\sigma ^\gamma }}\left( x \right) - f_\sigma ^\gamma + \left( {{{\left( {\omega _{g_\sigma ^\gamma }^*} \right) }^T}{\varphi _{g_\sigma ^\gamma }}\left( x \right) - g_\sigma ^\gamma } \right) u_\sigma ^\gamma . \end{aligned}$$

### Remark 1

Unknown functions $f_\sigma ^\gamma \left( x \right)$ and $g_\sigma ^\gamma \left( x \right)$ can be represented by RBFNN functions with different numbers of nodes which depends on the approximation error. The numbers of nodes will be increased if the approximation error requirement for the system’s stability is not satisfied. The upper limit for the approximation error will be given in next section.

**Controller design**. A dual switching tracking system is constructed to track reference signal ${y_d}$ and its derivatives ${y_d}^{\left( 1 \right) }, \ldots ,{y_d}^{\left( n \right) }$ which were generated by uncertain dual switching system (1). RBFNN functions are applied to approximate the unknown functions $f_\sigma ^\gamma \left( x \right)$ and $g_\sigma ^\gamma \left( x \right)$. The adaptive controller is constructed as

8 $$\begin{aligned} u_\sigma ^\gamma = {\left( {{\hat{g}}_\sigma ^\gamma \left( x \right) } \right) ^{ - 1}}\left( { - {\hat{f}}_\sigma ^\gamma \left( x \right) + \tau _\sigma ^\gamma } \right) \end{aligned}$$

where $\tau _\sigma ^\gamma = {y_d}^{\left( n \right) } + \alpha _{\sigma n}^\gamma \left( {{y_d}^{\left( {n - 1} \right) } - {y^{\left( {n - 1} \right) }}} \right) + \cdots + \alpha _{\sigma 1}^\gamma \left( {{y_d} - y} \right)$. Define $y = {x_1},{y^{\left( 1 \right) }} = {x_2}, \ldots ,{y^{\left( {n - 1} \right) }} = {x_n}$ , $x = {\left( {{x_1}, \ldots ,{x_n}} \right) ^T}$ and ${x_d} = {\left( {{y_d},y_d^{\left( 1 \right) }, \ldots ,y_d^{\left( {n - 1} \right) }} \right) ^T}$. The tracking error $e = {x_d} - x = {\left( {{y_d} - y, \ldots ,y_d^{\left( {n - 1} \right) } - {y^{\left( {n - 1} \right) }}} \right) ^T}$ is computed as follows:

9 $$\begin{aligned} {\dot{e} }= Ae + B\left( {y_d^{\left( n \right) } - {y^{\left( n \right) }}} \right) = Ae + B\left( {y_d^{\left( n \right) } - \left( {f_\sigma ^\gamma \left( x \right) + g_\sigma ^\gamma \left( x \right) u_\sigma ^\gamma } \right) } \right) . \end{aligned}$$

where $$A = \left[ {\begin{array}{*{20}{c}}0&{}{{I_{n - 1}}}\\ 0&{}0 \end{array}} \right] $$, $B = {\left( {0, \ldots ,1} \right) ^T}$. Combining Eqs. (6)–(8), the error system can be transformed as

10 $$\begin{aligned} \begin{aligned} {\dot{e}}&= Ae + B\left( {y_d^{\left( n \right) } - \left( {{\hat{f}}_\sigma ^\gamma \left( x \right) + {\hat{g}}_\sigma ^\gamma \left( x \right) u_\sigma ^\gamma - W_\sigma ^\gamma } \right) } \right) \\&= Ae + B\left( {y_d^{\left( n \right) } - {y_d}^{\left( n \right) } - \alpha {{_\sigma ^\gamma }_n}\left( {{y_d}^{\left( {n - 1} \right) } - {y^{\left( {n - 1} \right) }}} \right) - \cdots - \alpha {{_\sigma ^\gamma }_1}\left( {{y_d} - y} \right) + W_\sigma ^\gamma } \right) \\&= {\bar{A}}_\sigma ^\gamma e + BW_\sigma ^\gamma \\&= {\bar{A}}_\sigma ^\gamma e + B\left( {{{\left( {{\omega _{f_\sigma ^\gamma }} - \omega _{f_\sigma ^\gamma }^*} \right) }^T}{\varphi _{f_\sigma ^\gamma }}\left( x \right) + {{\left( {{\omega _{g_\sigma ^\gamma }} - \omega _{g_\sigma ^\gamma }^*} \right) }^T}{\varphi _{g_\sigma ^\gamma }}\left( x \right) u_\sigma ^\gamma } \right) + B\left( {{\varepsilon _{\min }}} \right) _\sigma ^\gamma \end{aligned} \end{aligned}$$

where $${\bar{A}}_\sigma ^\gamma = \left[ {\begin{array}{*{20}{c}} 0&{}A\\ { - \alpha {{_\sigma ^\gamma }_1}}&{}{ \cdots - \alpha {{_\sigma ^\gamma }_n}} \end{array}} \right] $$, $${\bar{B}} = \left[ {\begin{array}{*{20}{c}} 0\\ B \end{array}} \right] $$. By defining ${{\tilde{\omega }} _{f_\sigma ^\gamma }} = {\omega _{f_\sigma ^\gamma }} - \omega _{f_\sigma ^\gamma }^*$ and ${{{\tilde{\omega }} }_{g_\sigma ^\gamma }} = {\omega _{g_\sigma ^\gamma }} - \omega _{g_\sigma ^\gamma }^*$. The tracking error in (10) can be rewritten as follows:

11 $$\begin{aligned} {\dot{e}} = {\bar{A}}_\sigma ^\gamma e + B\left[ {{{{\tilde{\omega }} }_{f_\sigma ^\gamma }}^T{\varphi _{f_\sigma ^\gamma }}\left( x \right) + {{{\tilde{\omega }} }_{g_\sigma ^\gamma }}^T{\varphi _{g_\sigma ^\gamma }}\left( x \right) u_\sigma ^\gamma } \right] + B\left( {{\varepsilon _{\min }}} \right) _\sigma ^\gamma \end{aligned}$$

Our main goal is to investigate the GAS a.s. and ES a.s. properties of the dual switching nonlinear continuous time system (11). When the tracking error is stable, the uncertain DSS (1) approximated by using RBF neural network can track the original uncertainty system. The definitions of GAS a. s. and ES a. s.^20^ are given.

### Definition 1

The dual switching nonlinear continuous-time system is said to be

1. **globally asymptotically stable almost surely**, if the following two properties are verified simultaneously:
  SP1) for arbitrary $\varepsilon > 0$ , there exists a $\delta \left( \varepsilon \right) > 0$ such that when the system initial status ${x_0}$ satisfies $\left\| {{x_0}} \right\| < \delta \left( \varepsilon \right)$ , $P\left\{ {{{\sup }_{t \ge 0}}\left\| {x\left( t \right) } \right\| < \varepsilon } \right\} = 1$;
  SP2) for arbitrary $r > 0$ and ${\hat{\varepsilon }} > 0$ , there exists a $T\left( {r,{\hat{\varepsilon }} } \right) \ge 0$ such that when $\left\| {{x_0}} \right\| < r$ , $P\left\{ {{{\sup }_{t \ge T\left( {r,{\hat{\varepsilon }} } \right) }}\left\| {x\left( t \right) } \right\| < {\hat{\varepsilon }} } \right\} = 1$.
2. **almost surely exponentially stable**, if for all ${x_0} \in {R^n}$, $\mathrm{P}\left\{ {\mathop {\lim }\nolimits _{t \rightarrow \infty } \sup \frac{1}{t}\log \left\| {x\left( {t,{x_0}} \right) } \right\| < 0} \right\} = 1$.

### Lemma 1

^21^ *If* $${\alpha _1} \in \mathrm{K}$$ *and* $$\int _0^\infty {{\alpha _1}\left( {\left\| {x\left( t \right) } \right\| } \right) dt} < \infty $$ *a.s.*, $${\lim _{t \rightarrow \infty }}x\left( t \right) = 0$$ *a.s.*

## Results

Sufficient conditions for the dual switching system (11) are given by the energy attenuation theory and the Lyapunov function method.

### Theorem 1

*Consider the dual switching nonlinear continuous-time system* (11). *Suppose that there exist continuously differentiable functions* $V_i^{\left[ j \right] }\left( \cdot \right) :\;{R^n} \rightarrow {R^ + }$, $\;i \in N$ , $j \in \mathrm{M}$ , *functions* ${\alpha _1},{\alpha _2} \in {\mathrm{K}_\infty }$, *and constants* ${c_1},{c_2},{c_3}$ *as defined in the proof, such that the following conditions hold*:

1. $${\alpha _1}\left( {\left\| e \right\| } \right) \le V_i^{\left[ j \right] }\left( e \right) \le {\alpha _2}\left( {\left\| e \right\| } \right) ,\forall e \in {R^n}$$
2. $${\left( {{\bar{A}}_i^{\left[ j \right] }} \right) ^T}P_i^{\left[ j \right] } + P_i^{\left[ j \right] }{\bar{A}}_i^{\left[ j \right] } = - Q_i^{\left[ j \right] } < 0$$
3. $$\left( {{\varepsilon _{\min }}} \right) _i^{^{\left[ j \right] }} < {{{c_3}{c_2}} / {{c_1}}}$$

*Then, the system* (11) *is GAS a.s. under the switching strategy (H4)*:

(H4) $$\left\{ \begin{array}{l} {t_0} = 0\\ {\gamma _0} = \mathop {\arg \min \;}\nolimits _{j \in M} E\left( {\left| {\left( {{\varepsilon _{\min }}} \right) _{\sigma \left( {{t_0},j} \right) }^{\left[ j \right] }} \right| } \right) \\ \vdots \\ {t_k} = \inf \left\{ {t> {t_{k - 1}}|E\left( {\left| {\left( {{\varepsilon _{\min }}} \right) _{\sigma \left( {{t_{k - 1}},{\gamma _{k - 1}}} \right) }^{{\gamma _{k - 1}}}} \right| } \right) > E\left( {\left| {\left( {{\varepsilon _{\min }}} \right) _{\sigma \left( {t,j} \right) }^{\left[ j \right] }} \right| } \right) } \right\} \\ {\gamma _k} = \mathop {\arg \min \;}\nolimits _{j \in M} E\left( {\left| {\left( {{\varepsilon _{\min }}} \right) _{\sigma \left( {t,j} \right) }^{\left[ j \right] }} \right| } \right) \end{array} \right. $$

### *Proof*

**Figure 2:**
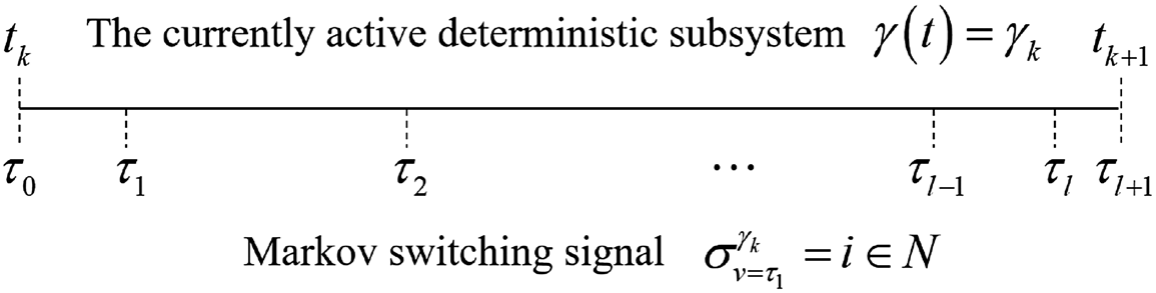
Stochastic switching timing diagram.

Define the Lyapunov function $V_i^{\left[ j \right] }(e(t)) = {e^T}(t)P_i^{\left[ j \right] }e(t) + {1 / 2}{{{\tilde{\omega }} }_{f_i^{\left[ j \right] }}}^T(t){({\Gamma _{f_i^{\left[ j \right] }}})^{ - 1}}{{{\tilde{\omega }} }_{f_i^{\left[ j \right] }}}(t) + {1 / 2}{{{\tilde{\omega }} }_{g_i^{\left[ j \right] }}}^T(t){({\Gamma _{g_i^{\left[ j \right] }}})^{ - 1}}{{\tilde{\omega } }_{g_i^{\left[ j \right] }}}(t)$. Suppose that $\gamma \left( t \right) = {\gamma _k} \in M$ for $t \in \left[ {{t_k},{t_{k + 1}}} \right)$. And there is no deterministic switching over this interval. $\left\{ {{\tau _0},{\tau _1}, \ldots ,{\tau _l}} \right\}$ is the stochastic switching time sequence of the Markov switching signal $\sigma \left( {t,{\gamma _k}} \right)$ over the interval $\left[ {{t_k},{t_{k + 1}}} \right)$ , shown in Fig. 2. The stochastic switching signal $\sigma \left( {t,{\gamma _k}} \right)$ is denoted by $\sigma _v^{{\gamma _k}} \in N$ for $t \in \left[ {{\tau _v},{\tau _{v + 1}}} \right)$, $v \in \left\{ {0,1,2, \ldots ,l} \right\} ,l < \infty$ .

The derivative of $V_i^{\left[ j \right] }(e(t))$ over the interval $\left[ {{t_k},{t_{k + 1}}} \right)$ is

12 $$\begin{aligned} \begin{aligned} {\dot{V}}_i^{^{{\gamma _k}}}(e(t)) =\, &{{\dot{e}}^T}(t)P_i^{^{{\gamma _k}}}e(t) + {e^T}(t)P_i^{^{{\gamma _k}}}{\dot{e}}(t) + \frac{1}{2} {{{\dot{\tilde{\omega }}} }_{f_i^{^{{\gamma _k}}}}}^T(t){({\Gamma _{f_i^{^{{\gamma _k}}}}})^{ - 1}}{{{\tilde{\omega }} }_{f_i^{^{{\gamma _k}}}}}(t) + \frac{1}{2}{{{\tilde{\omega }} }_{f_i^{^{{\gamma _k}}}}}^T(t){({\Gamma _{f_i^{^{{\gamma _k}}}}})^{ - 1}}{{{\dot{\tilde{\omega }}} } _{f_i^{^{{\gamma _k}}}}}(t) \\&+\frac{1}{2}{{{\dot{\tilde{\omega }}} }_{g_i^{^{{\gamma _k}}}}}^T(t){({\Gamma _{g_i^{^{{\gamma _k}}}}})^{ - 1}}{{\tilde{\omega }}_{g_i^{^{{\gamma _k}}}}}(t) + \frac{1}{2}{{{\tilde{\omega }} }_{g_i^{^{{\gamma _k}}}}}^T(t){({\Gamma _{g_i^{^{{\gamma _k}}}}})^{ - 1}}{{{\dot{\tilde{\omega }}}}_{g_i^{^{{\gamma _k}}}}}(t). \end{aligned} \end{aligned}$$

By combining this with (7), the Lyapunov function differential form is computed, as follows:

13 $$\begin{aligned} \begin{aligned} {\dot{V}}_i^{^{{\gamma _k}}}(e(t)) =\, &{e^T}(t){\left( {{\bar{A}}_i^{^{{\gamma _k}}}} \right) ^T}P_i^{^{{\gamma _k}}}e(t) + {e^T}(t)P_i^{^{{\gamma _k}}}{\bar{A}}_i^{^{{\gamma _k}}}e\left( t \right) + \left( {{{{\tilde{\omega }} }_{f_i^{^{{\gamma _k}}}}}^T(t){\varphi _{f_i^{^{{\gamma _k}}}}}(x) + {{{\tilde{\omega }} }_{g_i^{^{{\gamma _k}}}}}^T(t){\varphi _{g_i^{^{{\gamma _k}}}}}(x)u_i^{^{{\gamma _k}}}(x)} \right) {B^T}P_i^{^{{\gamma _k}}}e(t) \\&+ \left( {{\varepsilon _{\min }}} \right) _i^{^{{\gamma _k}}}{B^T}P_i^{^{{\gamma _k}}}e\left( t \right) + {e^T}(t)P_i^{^{{\gamma _k}}}B\left( {{{{\tilde{\omega }} }_{f_i^{^{{\gamma _k}}}}}^T(t){\varphi _{f_i^{^{{\gamma _k}}}}}(x) + {{{\tilde{\omega }} }_{g_i^{^{{\gamma _k}}}}}^T(t){\varphi _{g_i^{^{{\gamma _k}}}}}(x)u_i^{^{{\gamma _k}}}(x)} \right) + {e^T}\left( t \right) P_i^{^{{\gamma _k}}}B\left( {{\varepsilon _{\min }}} \right) _i^{^{{\gamma _k}}} \\&+ \frac{1}{2}{{{\dot{\omega }} }_{f_i^{^{{\gamma _k}}}}}^T(t){({\Gamma _{f_i^{^{{\gamma _k}}}}})^{ - 1}}{{{\tilde{\omega }} }_{f_i^{^{{\gamma _k}}}}}\left( t \right) + \frac{1}{2}{{{\tilde{\omega }} }_{f_i^{^{{\gamma _k}}}}}^T(t){({\Gamma _{f_i^{^{{\gamma _k}}}}})^{ - 1}}{{{\dot{\omega }} }_{f_i^{^{{\gamma _k}}}}}\left( t \right) \\&+ \frac{1}{2}{{{\dot{\omega }} }_{g_i^{^{{\gamma _k}}}}}^T(t){({\Gamma _{g_i^{^{{\gamma _k}}}}})^{ - 1}}{{{\tilde{\omega }} }_{g_i^{^{{\gamma _k}}}}}(t) + \frac{1}{2}{{{\tilde{\omega }} }_{g_i^{^{{\gamma _k}}}}}^T(t){({\Gamma _{g_i^{^{{\gamma _k}}}}})^{ - 1}}{{{\dot{\omega }} }_{g_i^{^{{\gamma _k}}}}}(t). \end{aligned} \end{aligned}$$

We define $\omega _i^{[j]} = ( {{\omega _{f_i^{[j]}}},{\omega _{g_i^{[j]}}}} )$, ${\Psi _{f_i^{[j]}}} = {e^T}P_i^{[j]}B\varphi _{f_i^{[j]}}^T(x)$ , ${\Psi _{g_i^{[j]}}} = {e^T}P_i^{[j]}B{\varphi _{g_i^{[j]}}}(x)u_i^{[j]}(x)$ , $\tilde{\omega } _i^{[j]} = ( {{{{\tilde{\omega }} }_{f_i^{[j]}}},{{\tilde{\omega } }_{g_i^{[j]}}}} )$, $\Psi _i^{[j]} = \left( {{\Psi _{f_i^{[j]}}},{\Psi _{g_i^{[j]}}}} \right)$, $\Gamma _i^{[j]} = diag\{ {{\Gamma _{f_i^{[j]}}},{\Gamma _{g_i^{[j]}}}} \}$. The adaptive law is designed as

14 $$\begin{aligned} \left\{ \begin{array}{l} {\dot{\omega }} _i^{[j]} = - 2\Gamma _i^{[j]}{\left( {\Psi _i^{[j]}} \right) ^T}\\ {\left( {{\tilde{\omega }} _i^{[j]}} \right) ^T}{\left( {\Gamma _i^{[j]}} \right) ^{ - 1}}({\dot{\omega }} _i^{[j]} + \Gamma _i^{[j]}{\left( {\Psi _i^{[j]}} \right) ^T}) \le 0 \end{array} \right. \end{aligned}$$

Thus, combining (8), (H2), and ${c_1} = 2\mathop {\max }\nolimits _{e \in E} \left\| e \right\| \times \left\| {P_i^{[j]}B} \right\|$ yields the following Lyapunov function differential simplified form

15 $$\begin{aligned} \dot{V}_i^{^{{\gamma _k}}}(e(t)) \le - {e^T}(t)Q_i^{^{{\gamma _k}}}e\left( t \right) + {c_1}\left( {{\varepsilon _{\min }}} \right) _i^{^{{\gamma _k}}} \le - {e^T}(t)Q_i^{^{{\gamma _k}}}e\left( t \right) + {c_1}\left| {\left( {{\varepsilon _{\min }}} \right) _i^{^{{\gamma _k}}}} \right| \end{aligned}$$

By Dynkin’s formula, computing the expected value of both sides of inequality (15) yields the following:

16 $$\begin{aligned}{}&E\left[ {V_l^{{\gamma _k}}(e(t)) - V_0^{{\gamma _k}}(e({t_k}))} \right] \nonumber \\&\quad = E\left[ {\int _{{\tau _l}}^t {\dot{V}_l^{^{{\gamma _k}}}\left( {e\left( s \right) } \right) ds} + \int _{{\tau _{l - 1}}}^{{\tau _l}} {\dot{V}_{l - 1}^{^{{\gamma _k}}}\left( {e\left( s \right) } \right) ds} + \cdots \int _{{\tau _0}}^{{\tau _1}} {\dot{V}_0^{^{{\gamma _k}}}\left( {e\left( s \right) } \right) ds} } \right] \nonumber \\&\quad \le - \left( {{\lambda _{\min }}\left( {Q_i^{^{{\gamma _k}}}} \right) } \right) E\left[ {\int _{{t_k}}^t {{e^T}\left( s \right) e\left( s \right) ds} } \right] + {c_1}E\left[ {\int _{{\tau _l}}^t {\left| {\left( {{\varepsilon _{\min }}} \right) _l^{^{{\gamma _k}}}} \right| ds} + \int _{{\tau _{l - 1}}}^{{\tau _l}} {\left| {\left( {{\varepsilon _{\min }}} \right) _{l - 1}^{^{{\gamma _k}}}} \right| ds} + \cdots \int _{{\tau _0}}^{{\tau _1}} {\left| {\left( {{\varepsilon _{\min }}} \right) _0^{^{{\gamma _k}}}} \right| ds} } \right] \nonumber \\&\quad \le - \left( {{\lambda _{\min }}\left( {Q_i^{^{{\gamma _k}}}} \right) } \right) E\left[ {\int _{{t_k}}^t {{e^T}\left( s \right) e\left( s \right) ds} } \right] + {c_1}\int _{{t_k}}^t {E\left[ {\left| {\left( {{\varepsilon _{\min }}} \right) _{\sigma \left( {s,{\gamma _k}} \right) }^{{\gamma _k}}} \right| } \right] ds} \end{aligned}$$

At the switching moment ${t_k}$ , with the switching rules in (H4), this becomes the following:

17 $$\begin{aligned}{}&E\left[ {V_{\sigma \left( {t,{\gamma _k}} \right) }^{{\gamma _k}}(e(t)) - V_{\sigma \left( {{t_{k - 1}},{\gamma _{k - 1}}} \right) }^{{\gamma _{k - 1}}}(e({t_{k - 1}}))} \right] \nonumber \\&\quad \le E\left[ {V_{\sigma \left( {t,{\gamma _k}} \right) }^{{\gamma _k}}(e(t)) - V_{\sigma \left( {{t_k},{\gamma _k}} \right) }^{{\gamma _k}}(e({t_k}))} \right] + E\left[ {V_{\sigma \left( {{t_k},{\gamma _{k - 1}}} \right) }^{{\gamma _{k - 1}}}(e({t_k})) - V_{\sigma \left( {{t_{k - 1}},{\gamma _{k - 1}}} \right) }^{{\gamma _{k - 1}}}(e({t_{k - 1}}))} \right] \nonumber \\&\quad \le - \left( {{\lambda _{\min }}\left( {Q_i^{^{{\gamma _k}}}} \right) } \right) E\left[ {\int _{{t_k}}^t {{e^T}\left( s \right) e\left( s \right) ds} } \right] + {c_1}\int _{{t_k}}^t {E\left[ {\left| {\left( {{\varepsilon _{\min }}} \right) _{\sigma \left( {s,{\gamma _k}} \right) }^{{\gamma _k}}} \right| } \right] ds} \nonumber \\&\qquad - \left( {{\lambda _{\min }}\left( {Q_i^{^{{\gamma _{k\mathrm{{ - 1}}}}}}} \right) } \right) E\left[ {\int _{{t_{k\mathrm{{ - 1}}}}}^{{t_k}} {{e^T}\left( s \right) e\left( s \right) ds} } \right] + {c_1}\int _{{t_{k - 1}}}^{{t_k}} {E\left[ {\left| {\left( {{\varepsilon _{\min }}} \right) _{\sigma \left( {s,{\gamma _{k - 1}}} \right) }^{{\gamma _{k - 1}}}} \right| } \right] ds} \end{aligned}$$

Applying the above inequality over $\left[ {0,t} \right)$ yields

18 $$\begin{aligned} \begin{aligned}{}&E\left[ {V_{\sigma \left( {t,{\gamma _k}} \right) }^{{\gamma _k}}(e(t)) - V{{_{\sigma \left( {{t_0},{\gamma _0}} \right) }^{{\gamma _0}}}^{{\gamma _0}}}(e({t_0}))} \right] \\&\quad \le - \left( {{\lambda _{\min }}\left( {Q_i^{^{{\gamma _k}}}} \right) } \right) E\left[ {\int _{{t_k}}^t {{e^T}\left( s \right) e\left( s \right) ds} } \right] - \left( {{\lambda _{\min }}\left( {Q_i^{^{{\gamma _{k\mathrm{{ - 1}}}}}}} \right) } \right) E\left[ {\int _{{t_{k\mathrm{{ - 1}}}}}^{{t_k}} {{e^T}\left( s \right) e\left( s \right) ds} } \right] \\&\qquad + {c_1}\int _{{t_k}}^t {E\left[ {\left| {{{\left( {{\varepsilon _{\min }}} \right) }}_{\sigma \left( {s,{\gamma _k}} \right) }^{{\gamma _k}}} \right| } \right] ds + \cdots + } {c_1}\int _0^{{t_1}} {E\left[ {\left| {\left( {{\varepsilon _{\min }}} \right) _{\sigma \left( {s,{\gamma _0}} \right) }^{{\gamma _0}}} \right| } \right] ds} \end{aligned} \end{aligned}$$

The expression (18) can be simplified with the definition ${c_2} = \mathop {\max }\nolimits _{i \in N,j \in M} {e^T}\left( t \right) P_i^{^{\left[ j \right] }}e\left( t \right)$, ${c_3} = \mathop {\min }\nolimits _{i \in N,j \in M} {{{\lambda _{\min }}(Q_i^{^{\left[ j \right] }})} / {{\lambda _{\max }}(P_i^{^{\left[ j \right] }})}}$, ${\bar{\lambda }} = \mathop {\min }\nolimits _{i \in N,j \in M} {\lambda _{\min }}\left( {Q_i^{^{\left[ j \right] }}} \right)$, ${\bar{\eta }} = \mathop {\max }\nolimits _{i \in N,j \in M} {\lambda _{\min }}\left( {P_i^{^{\left[ j \right] }}} \right)$, and thus the upper limit of the optimal approximation error

19 $$\begin{aligned} \left( {{\varepsilon _{\min }}} \right) _i^{^{\left[ j \right] }}< {{\left( {{{{\lambda _{\min }}(Q_i^{^{\left[ j \right] }})} / {{\lambda _{\max }}(P_i^{^{\left[ j \right] }})}}} \right) {e^T}\left( t \right) e\left( t \right) } / {{c_1}}} < {{{c_3}{c_2}} / {{c_1}}} \end{aligned}$$

is given here. Equation (18) can be rewritten as

20 $$\begin{aligned} E\left[ {V_{\sigma \left( {t,{\gamma _k}} \right) }^{{\gamma _k}}(e(t)) - V_{\sigma \left( {{t_0},{\gamma _0}} \right) }^{{\gamma _0}}(e({t_0}))} \right] \le - {\bar{\lambda }} E\left[ {\int _0^t {{e^T}\left( s \right) e\left( s \right) ds} } \right] + {c_3}\bar{\eta }E\left[ {\int _0^t {{e^T}\left( s \right) e\left( s \right) ds} } \right] . \end{aligned}$$

After rewriting the previous equation again, the proof-friendly form (21) is obtained.

21 $$\begin{aligned}{}&\left( {{\bar{\lambda }} - {c_3}{\bar{\eta }} } \right) E\left[ {\int _0^t {{e^T}\left( t \right) e\left( t \right) ds} } \right] \le E\left[ {V_{\sigma \left( {{t_0},{\gamma _0}} \right) }^{{\gamma _0}}(e({t_0}))} \right] - E\left[ {V_{\sigma \left( {t,{\gamma _k}} \right) }^{{\gamma _k}}(e(t))} \right] \le E\left[ {V_{\sigma \left( {{t_0},{\gamma _0}} \right) }^{{\gamma _0}}(e({t_0}))} \right] \end{aligned}$$

22 $$\begin{aligned}{}&E\left[ {\int _0^t {{e^T}\left( s \right) e\left( s \right) ds} } \right] \le \frac{{E\left[ {V_{\sigma \left( {{t_0},{\gamma _0}} \right) }^{{\gamma _0}}(e({t_0}))} \right] }}{{\left( {{\bar{\lambda }} - {c_3}{\bar{\eta }} } \right) }} = \mathrm{Z}\left( {{e_0},{\gamma _0}} \right) . \end{aligned}$$

Obviously, $E\left[ {\int _0^t {{e^T}\left( s \right) e\left( s \right) ds} } \right]$ is bounded. Then, by Lemma 1, $\mathop {\lim }\nolimits _{t \rightarrow \infty } \left\| {e\left( t \right) } \right\| = 0$ can be obtained, which satisfies SP1) in Definition 1.

We now verify SP2) of Definition 1. We select $\delta \left( {\hat{\varepsilon }} \right) = \min \left\{ {\alpha _2^{ - 1}\left( {{\alpha _1}\left( {{\hat{\varepsilon }} } \right) } \right) } \right\}$ . By transforming (21) with (H1), ${\alpha _1}\left( {\left\| {e\left( t \right) } \right\| } \right) \le E\left[ {V_{\sigma \left( {t,{\gamma _k}} \right) }^{{\gamma _k}}(e(t))} \right] \le E\left[ {V_{\sigma \left( {{t_0},{\gamma _0}} \right) }^{{\gamma _0}}(e({t_0}))} \right] \le {\alpha _2}\left( {\left\| {{e_0}} \right\| } \right)$ can be obtained. Then, $\left\| {{e_0}} \right\| < \delta \left( {{\hat{\varepsilon }} } \right)$ implies that for any $t \in \left[ {0,T\left( {1,{\hat{\varepsilon }} } \right) } \right]$, $\left\| {e\left( t \right) } \right\| \le \alpha _1^{ - 1}\left[ {{\alpha _2}\left( {\left\| {{e_0}} \right\| } \right) } \right] \le {\hat{\varepsilon }}$ . Furthermore, the SP2) property guarantees that with the previous choice of $\delta \left( {{\hat{\varepsilon }} } \right)$ and ${e_0}$, we have ${\sup _{t \ge \mathrm{T}\left( {1,\hat{\varepsilon }} \right) }}\left\| {e\left( t \right) } \right\| < \hat{\varepsilon }$ on a set of full measure. Therefore, $\left\| {{e_0}} \right\| < \delta \left( {{\hat{\varepsilon }} } \right)$ implies ${\sup _{t \ge 0}}\left\| {e\left( t \right) } \right\| < {\hat{\varepsilon }}$ a.s. Because of the arbitrariness of ${\hat{\varepsilon }}$, the SP2) property of Definition 1 follows. We conclude that the dual switching nonlinear continuous-time system (11) is GAS a.s.

### Remark 2

Many scholars have proposed different analysis methods for the stability analysis of dual switching systems, such as Bolzern^8^ and Yang^4,7,9^. Unlike the above researches, the research object of this paper is a dual switching nonlinear system with uncertain functions. The control mechanism is more complex, the switching rule design for the switching rules are higher, and the stability sufficient conditions for the system are more stringent.

### Remark 3

$V_i^{\left[ j \right] }(e(t))$ denotes the Lyapunov function of the i-th mode of the j-th deterministic subsystem of the error tracking system (11). If the dual switching system has *M* deterministic subsystems, each with *N* stochastic subsystems, then $M \times N$ Lyapunov functions need to be defined.

### Remark 4

Importantly, despite the switching between subsystems, transient stability can be guaranteed at the switching instant. The transient performance can be guaranteed because of the boundary of the tracking error.

### Remark 5

Dual switching system (11) is composed of multiple Markovian jump subsystems and a deterministic switching strategy. To determine the influence of randomness, we introduce $E( {| {( {{\varepsilon _{\min }}} )_{\sigma ( {t,j} )}^{[ j ]}} |} )$ to design switching rules. Combined with the Lyapunov function method, an adaptive control law is designed to adjust the weights of the RBFNN online to obtain a better tracking effect.

When (H1) in Theorem 1 is further restricted, GAS a.s. is upgraded to ES a.s. The conclusion and proof are described in the following corollary.

### Corollary 1

*Consider the dual switching nonlinear continuous-time system* (11). *There exist constants and such that the following holds*:

1. $$c{\left\| e \right\| ^p} < V_i^{[j]},\forall e \in {R^n}$$

*If (H2) and (H3) in Theorem* 1*hold, then system *(11) *is ES a.s. under the control of switching strategy in* (H4).

### *Proof*

It can be verified with (18) and (H1’) that for $\forall t > 0$, $c{\left\| {e\left( t \right) } \right\| ^p}< \mathrm{E}\left[ {V_{\sigma \left( {t,\gamma \left( t \right) } \right) }^{\left[ {\gamma \left( t \right) } \right] }\left( {e\left( t \right) } \right) } \right] < \mathrm{E}\left[ {V_{\sigma \left( {0,{\gamma _0}} \right) }^{\left[ {{\gamma _0}} \right] }\left( {{e_0}} \right) } \right]$. That is, $\frac{1}{t}\log \left\| {e\left( t \right) } \right\| < \frac{1}{{tp}}\left( {\log \mathrm{E}\left[ {V_{\sigma \left( {0,{\gamma _0}} \right) }^{\left[ {{\gamma _0}} \right] }\left( {{e_0}} \right) } \right] - \log c} \right)$ . Thus, $\mathop {\lim }\nolimits _{t \rightarrow \infty } \sup \frac{1}{t}\log \left\| {e\left( t \right) } \right\| < 0$ . According to Definition 1, the dual switching nonlinear continuous-time system (11) of the tracking error is ES a.s. This completes the proof. $\square$

### Remark 6

If the dual switching system is ES a.s., the system state distribution is exponentially attenuated. The ES a.s. condition is stricter than the GAS a.s. condition, which is known from (H1’) in Corollary 1. In other words, if the dual switching nonlinear system is ES a.s., it must be GAS a.s., but not vice versa.

## Simulation

In this example, we show how the RBF neural networks approximate the unknown system and synthesize an adaptive controller to stabilize the tracking system. We consider a dual switching system with two subsystems, and each of them has two modes.

- Subsystem 1 has two modes that are defined as
  mode 1: $$\left\{ \begin{array}{l} \dot{x} = Ax + B\left( {f_1^{\left[ 1 \right] }\left( x \right) + g_1^{\left[ 1 \right] }\left( x \right) u_1^{\left[ 1 \right] }} \right) \\ y = Cx \end{array} \right. $$
  mode 2: $$\left\{ \begin{array}{l} \dot{x} = Ax + B\left( {f_2^{\left[ 1 \right] }\left( x \right) + g_2^{\left[ 1 \right] }\left( x \right) u_2^{\left[ 1 \right] }} \right) \\ y = Cx \end{array} \right. $$
  The transition probability matrix of subsystem 1 is defined as $$tr1 = \left[ {\begin{array}{*{20}{c}} {0.4}&{}{0.6}\\ {0.5}&{}{0.5} \end{array}} \right] $$.
- Subsystem 2 has two modes that are defined as
  mode 1: $$\left\{ \begin{array}{l} \dot{x} = Ax + B\left( {f_1^{\left[ 2 \right] }\left( x \right) + g_1^{\left[ 2 \right] }\left( x \right) u_1^{\left[ 2 \right] }} \right) \\ y = Cx \end{array} \right. $$
  mode 2: $$\left\{ \begin{array}{l} \dot{x} = Ax + B\left( {f_2^{\left[ 2 \right] }\left( x \right) + g_2^{\left[ 2 \right] }\left( x \right) u_2^{\left[ 2 \right] }} \right) \\ y = Cx \end{array} \right. $$
  The transition probability matrix of this subsystem is defined as $$tr2 = \left[ {\begin{array}{*{20}{c}} {0.7}&{}{0.3}\\ {0.2}&{}{0.8} \end{array}} \right] $$.

**Figure 3:**
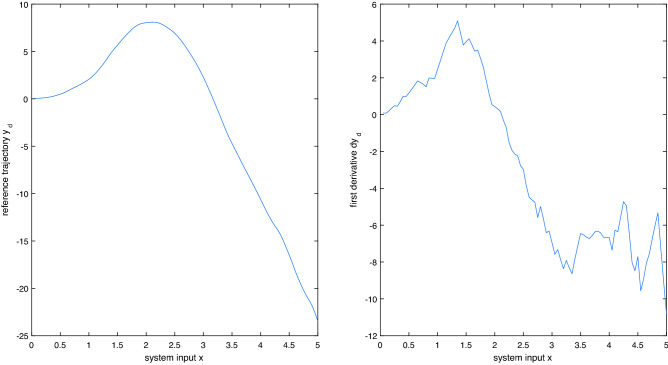
the reference signal ${y_d}$ and its derivatives ${y_d}^{\left( 1 \right) }$.

For uncertain dual switching nonlinear system (1), the functions $f_\sigma ^\gamma$ and $g_\sigma ^\gamma$ are unknown, but their historical data can be observed. Similarly, the reference signal ${y_d}$ and its derivatives ${y_d}^{\left( 1 \right) }$ are also observable , and they are shown in Fig. 3.

**Figure 4:**
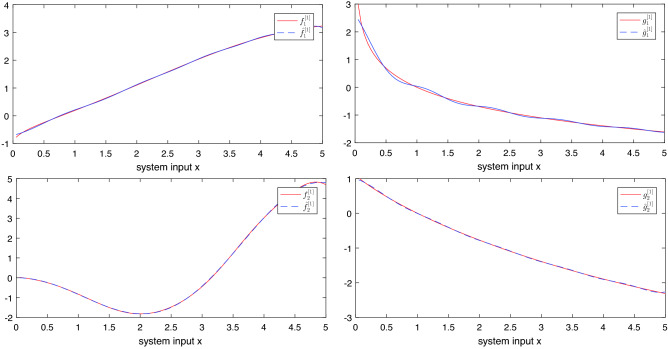
Uncertain functions and its RBFNN approximation values of subsystem 1.

**Figure 5:**
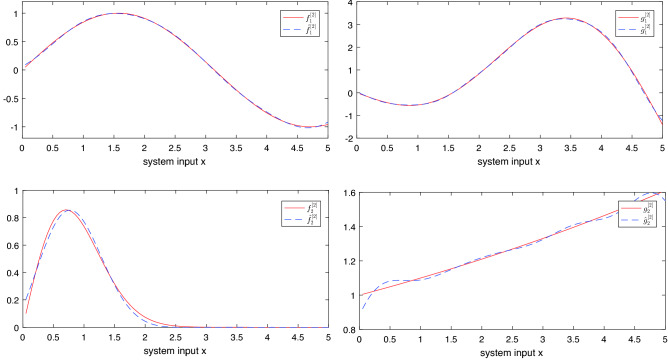
Uncertain functions and its RBFNN approximation values of subsystem 2.

**RBFNN approximation**. To fit the curves $f_\sigma ^\gamma$ and $g_\sigma ^\gamma$, Gaussian basis functions were selected. The upper limit of approximation error was set to 0.05. The RBFNN approximation result of the uncertain functions are shown in Figs. 4 and 5.

**Figure 6:**
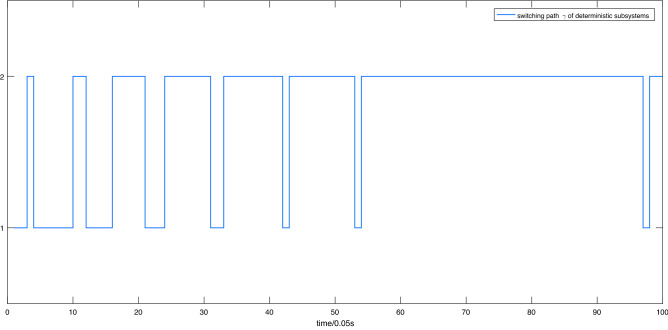
Switching path $\gamma$ of deterministic subsystems.

**Tracking trajectory produced by switching strategies**. We define $\alpha _{11}^{[1]} = \alpha _{11}^{[2]} = \alpha _{21}^{[1]} = \alpha _{21}^{[2]} = - 1$ , $\alpha _{12}^{[1]} = \alpha _{12}^{[2]} = \alpha _{22}^{[1]} = \alpha _{22}^{[2]} = - 2$ , and $$Q_i^{\left[ j \right] } = \left[ {\begin{array}{*{20}{c}} 1&{}0\\ 0&{}1 \end{array}} \right] $$, and then we can solve for $P_i^{\left[ j \right] }$. $$P_i^{\left[ j \right] } = \left[ {\begin{array}{*{20}{c}} {1.5}&{}{0.5}\\ {0.5}&{}{0.5} \end{array}} \right] $$ satisfies condition (H2) of the Theorem 1. The controller response can be calculated as $u_i^{[j]} = {\left( {{\hat{g}}_i^{[j]}\left( x \right) } \right) ^{ - 1}}\left( { - {\hat{f}}_i^{[j]}\left( x \right) + {y_d}^{\left( 2 \right) } - 2\left( {{y_d}^{\left( 1 \right) } - {y^{\left( 1 \right) }}} \right) - \left( {{y_d} - y} \right) } \right)$. Constants ${c_1},{c_2},{c_3}$ can be obtained as ${c_1} = 2*\mathop {\max }\nolimits _{e \in E,i \in N,j \in M} \left\| e \right\| \times \left\| {P_i^{[j]}B} \right\| = 2*2.1349 = \mathrm{{ 4}}\mathrm{{.2657}}$, ${c_2} = \mathop {\max }\nolimits _{i \in N,j \in M} \left( {{e^T}\left( t \right) P_i^{^{\left[ j \right] }}e\left( t \right) } \right) = \mathrm{{13}}\mathrm{{.0045}}$ and ${c_3} = \mathop {\min }\nolimits _{i \in N,j \in M} \left( {{{{\lambda _{\min }}(Q_i^{^{\left[ j \right] }})} / {{\lambda _{\max }}(P_i^{^{\left[ j \right] }})}}} \right) \mathrm{{ = 0}}\mathrm{{.5858}}$ by computation. Then, it can be concluded that the minimum network error $\left( {{\varepsilon _{\min }}} \right) _i^{^{\left[ j \right] }}$ of each mode in both subsystems satisfies (H3) in Theorem 1. The deterministic subsystem always switches to the one with minimum expected error under the control of the switching rule in (H4) to achieve a better tracking effect. The switching path is shown in Fig. 6.

**Figure 7:**
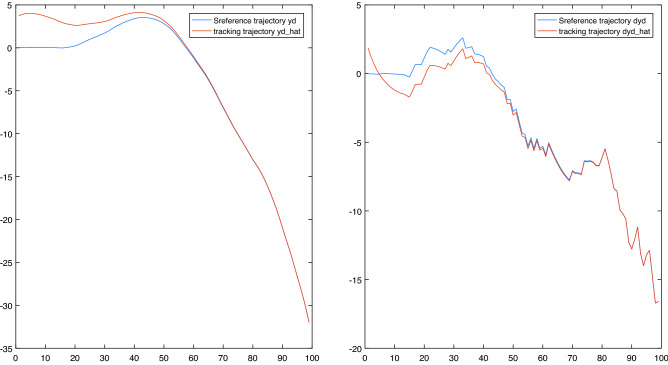
Tracking effect of reference trajectory.

Based on the above analysis, the dual switching system could track the original reference signal well under the switching strategy using the adaptive controller based on the neural network. The result is shown in Fig. 7.

**Figure 8:**
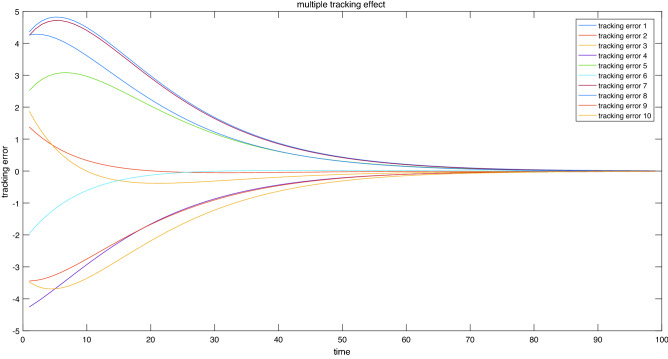
Multiple tracking effect.

**Multiple tracking effects of stochastic processes**. Constants $c = 1$ and $p = 1$ were selected, which satisfy (H1’) in Corollary 1. When the simulation was run several times with stochastic uncertainty of the subsystem, the dual switching system approximated by RBFNN could still track the reference trajectory well. The simulation results are shown in Fig. 8.

These simulation results indicate that the presented controller can effectively guarantee that the tracking error is GAS a.s. and ES a.s..

## Conclusion

In this article, an adaptiveneural network control issues for uncertain dual switching nonlinear systems has been investigated. Utilizing the properties of RBFNNs and the Lyapunov theorem, an adaptive controller is constructed under the pre-designed switching strategy. With the given controller, the GAS a.s. and the ES a.s. for the tracking error system is studied. Finally, the simulation results showed that the proposed method could track the state of an uncertain dual switching system accurately, and the feasibility and effectiveness were verified.

## Data Availability

All data generated or analysed during this study are included in this published article.
